# Properties of Nanohydroxyapatite Coatings Doped with Nanocopper, Obtained by Electrophoretic Deposition on Ti13Zr13Nb Alloy

**DOI:** 10.3390/ma12223741

**Published:** 2019-11-13

**Authors:** Michał Bartmański, Łukasz Pawłowski, Gabriel Strugała, Aleksandra Mielewczyk-Gryń, Andrzej Zieliński

**Affiliations:** 1 Faculty of Mechanical Engineering, Gdansk University of Technology, Narutowicza 11/12, 80-233 Gdańsk, Poland; lukasz.pawlowski@pg.edu.pl (Ł.P.); gabriel.strugala@pg.edu.pl (G.S.); andrzej.zielinski@pg.edu.pl (A.Z.); 2 Faculty of Applied Physics and Mathematics, Gdansk University of Technology, Narutowicza 11/12, 80-233 Gdańsk, Poland; alegryn@pg.edu.pl

**Keywords:** nanohydroxyapatite, nanocopper particles, titanium alloy

## Abstract

Nowadays, hydroxyapatite coatings are the most common surface modification of long-term implants. These coatings are characterized by high thickness and poor adhesion to the metallic substrate. The present research is aimed at characterizing the properties of nanohydroxyapatite (nanoHAp) with the addition of copper nanoparticle (nanoCu) coatings deposited on the Ti13Zr13Nb alloy by an electrophoresis process. The deposition of coatings was carried out for various amounts of nanoCu powder and various average particle sizes. Microstructure, topography, phase, and chemical composition were examined with scanning electron microscopy, atomic force microscopy, and X-ray diffraction. Corrosion properties were determined by potentiodynamic polarization technique in simulated body fluid. Nanomechanical properties were determined based on nanoindentation and scratch tests. The wettability of coatings was defined by the contact angle. It was proven that nanoHAp coatings containing nanocopper, compared to nanoHAp coatings without nanometals, demonstrated smaller number of cracks, lower thickness, and higher nanomechanical properties. The influence of the content and the average size of nanoCu on the quality of the coatings was observed. All coatings exhibited hydrophilic properties. The deposition of nanohydroxyapatite coatings doped with nanocopper may be a promising way to improve the antibacterial properties and mechanical stability of coatings.

## 1. Introduction

Titanium and its alloys have become the most advanced and most often proposed biomaterials for long-term load-bearing implants or their parts, such as dental implants, orthopedic implants, or maxillofacial implants. They demonstrate reasonable and sufficient mechanical properties except for wear resistance, their Young modulus is reasonably close to that of a bone, and they are characterized by high corrosion resistance followed by high biocompatibility and no adverse effects after implantation [1,2,3]. Nowadays, two surface features are commonly expected in modern implants: bioactivity and antimicrobial properties.

Bioactivity is assumed as an ability to create strong bonding between an implant and a bone in a short time after implantation. The most straightforward solutions include chemical etching of the surface with acids [2,4], alkalis [5,6], or hydrogen peroxide [7]. A more advanced treatment method is electrochemical oxidation resulting in nanotubular oxide layers [5,8] and micro-arc oxidation in electrolytes followed by tight and thick oxides of high roughness [9,10,11]. Ion implantation is also proposed [12,13]. The most attractive technique seems to be the deposition of different coatings such as chitosan [14,15,16,17], silicates [18], polyurethanes [19], and, much preferred, phosphates of varying chemical composition.

Phosphate coatings differ from each other in their chemical composition. Hydroxyapatites (HAp) [20,21,22,23,24,25], e.g., TCP [26,27], BCP [28,29] can be used. HAp coatings may be formed in microcrystalline form or as nanohydroxyapatites (nanoHAp) [8,16,30,31,32,33,34,35,36,37,38,39,40,41]. HAp or nanoHAp coatings may be chemically reformulated to replace calcium with such metals as: Mg [36,42,43], Fe [35], Zn [40,43,44,45,46,47], La/Cu [43], Sr [48,49], Sr/Mn [50], Sr/Cu [51], Ce [21,39], Cu [52,53,54,55] to improve the adhesion or antibacterial efficacy. Composite or hybrid coatings such as HAp or nanoHAp coatings implemented with TiO_2_ [10,11,56] and carbon nanotubes (CNTs) [38,40,57] are also investigated. Deposition is often preceded by oxidation of the surface to obtain nanostructures [17,20,24,25,34,58].

Phosphate coatings are obtained through different methods. They include electro-cathodic deposition [20,59,60,61], electrophoretic deposition [31,37,40,62], sol–gel deposition [61,63,64], biomimetic method [41,65,66], PVD magnetron sputtering [56,58,67,68], CVD [68,69], and laser ablation [70,71]. When strongly adhesive and uniform coatings are planned, the electrophoretic deposition (EPD) technique applied in this research is among a few of the most plausible methods resulting in coatings that are very thin and resistant to shear stress [20,23,31,37,38,40].

During the time of healing, an inflammatory condition is very grave, as it may result in implant rejection. Prolonged inflammation may also cause a decrease in the pH value—if it is accompanied by the occurrence of organic acids, severe titanium corrosion can occur [72,73]. Consequently, antibacterial properties become more and more critical in the process of coating design. These properties can be achieved by surface grafting, surface nanostructurization, or application of coatings [68]. Among such countermeasures, silver or nanosilver additives to coatings are most often described and recommended [17,22,30,40,49,53,54,55,74,75,76]. Copper or nanocopper additives [43,51,71] are considered in second place even if their efficacy and possible adverse effects are still under discussion.

Copper is involved in vital cell functions, it is present in metabolic processes and stimulates the activity of several enzymes. It also enhances the cross-linking of collagen and elastin of bones [77,78,79]. Metallic copper is efficient in wound healing in a better way than copper ions [14]. Its bacteriostatic and antibacterial effects are achieved by modifying cellular permeability and causing dysfunction and death of bacterial cells [79]. 

The antibacterial efficacy of copper was demonstrated for a variety of strains. The addition of nanoCu into HAp introduced an excellent effect against *Escherichia coli* and *Staphylococcus aureus* [71]. The Cu-implemented HAp demonstrated a reduction of cell ability for propagation of *E. coli*, *S. aureus*, and *Candida albicans* [52]. CaP cement implemented with Cu ions showed antibacterial effect against *E. coli*, *Pseudomonas aeruginosa,* and *Salmonella enteritidis* [80].

Copper and nanocopper are not widely applied in medicine for two reasons: bacterial efficacy and health risk. Nanocopper is usually used in medicine as a metallic element [51,52,71,81,82], or antibacterial agent in HAp [83] or nanoHAp coatings [81,84]. Copper is well accepted by the human body, but nanocopper may be toxic at higher doses. In [85], hepatotoxicity caused by copper ions formed by dissolution of nanocopper was observed at 4.5 μg/g of Cu^2+^. The acute toxicity of nanocopper was reported in some studies and was also said to damage the liver, kidneys, and spleen [86]. The safe content of copper in coatings can be estimated as 2 wt.% as concerns the compatibility [71]. On the other hand, CaP cement doped with ionic copper increased the viability of human E297 cells, some osteoblasts, and fibroblasts, and its high antibacterial efficacy was shown to be achieved at low cytotoxicity [77].

Nanohydroxyapatite (nanoHAp) coatings mean the hydroxyapatite coatings deposited from powder with an average grain size in nanometric range, up to 100 nm. The nanoHAp coatings doped with nanocopper have not been an object of many studies. Therefore, research presented here was conducted to characterize the physical, chemical, and mechanical properties of such coatings on titanium surface and, in particular, the effects of nanocopper content and nanoparticle mean size. The specific purpose is that there have been plenty of similar studies of nanosilver, much less on copper, and only few published in essential journals on nanocopper. The reason is that nanosilver has become totally safe and easy for commercialization, and effects of nanocopper (or nanogold) are still under discussion. We have proposed nanocopper, expecting that such element in this form will be necessary for an increase in adhesion of coatings, all the time subject to high stresses during implantation surgery (for whatever implant), and it may fortify the antibacterial effect without severe deterioration of biocompatibility. Ti13Zr13Nb alloy, the most suitable biomaterial for long-term implants due to its low Young’s modulus close to that of a bone, possessing no toxic elements in its composition, was deposited by the electrophoretic method. The hydroxyapatite coating was subject to thermal treatment, and then surface morphology, topography, chemical and phase composition, nanomechanical properties, and wettability were investigated.

## 2. Materials and Methods

### 2.1. Preparation of Specimens

Ti13Zr13Nb titanium alloy (SeaBird Metal Materials Co., Baoji, China) was used as a substrate. The chemical composition of the tested material is given in Table 1. Four-millimeter thick quadrant specimens were cut from a circular rod whose radius was 15 mm. The surface of the samples was subjected to wet grinding with SiC abrasive paper with the final gradation of #2000. The prepared material was cleaned and degreased by washing with isopropanol, placed in an ultrasonic washer (Sonic 3, Polsonic, Warsaw, Poland) in distilled water for 60 min and then immersed in 25% nitric acid for 10 min to remove the titanium dioxide layer [87]. The specimens were rinsed with distilled water before electrophoretic deposition.

### 2.2. Electrophoretic Deposition of Nanohydroxyapatite and Cu Nanoparticles

The electrophoretic deposition process was carried out in a suspension containing 100 mL of anhydrous (99.8%) ethyl alcohol (Sigma Aldrich, St. Louis, MO, United States) and 0.1 g of nanohydroxyapatite powder with average particle size 20 nm and 99.8% purity (MKnano, Missisaug, Canada). Two fractions of nanoCu were investigated: with average particles size 40 and 80 nm (Hongwu International Group Ltd., Guangzhou, China). The appropriate amount of nanoparticles was dispersed in ethanol in an ultrasonic cleaner (MKD Ultrasonic, Warsaw, Poland) for 60 min at room temperature to avoid the formation of agglomerates. Ti13Zr13Nb alloy was the cathode and platinum foil was the counter electrode. The distance between the parallel electrodes connected to the DC power supply (MCP/SPN-C, Shanghai MPC Corp., Shanghai, China) was about 10 mm. The deposition was carried out at 30 V for 2 min at room temperature. After deposition, the specimens were dried for 24 h at room temperature. Table 2 shows the designations of individual samples and characteristic process parameters.

### 2.3. Thermal Treatment

After deposition, the specimens with pure nanohydroxyapatite and nanohydroxyapatite coatings with copper nanoparticles were thermally treated in a tubular furnace (Protherm PC442, Ankara, Turkey) in vacuum at 800 °C for 120 min. The sintering process was carried out at the heating rate of 200 °C/h. Then, the specimens were cooled to room temperature in the furnace. The sintering of coated samples was conducted to increase the coating adhesion to the substrate, reduce its porosity, and change the crystal structure of nanoHAp [88]. 

### 2.4. Surface Analysis

Macroscopic images of the coatings’ surface were taken using a high-resolution digital camera (Nikon D7000, lens Tamron 90mm f/2.8 Macro, Nikon, Tokyo, Japan). The microstructures of the surface and cross-sections of deposited coatings were studied using scanning electron microscopy (SEM, JEOL JSM–7800 F, JEOL Ltd., Tokio, Japan ) at different magnifications. The surface topography was investigated by atomic force microscopy (NaniteAFM, Nanosurf AGLiestal, Switzerland) using non-contact mode at a force of 55 nN at an area of 80.4 × 80.4 µm. The surface roughness parameter Sa was determined with software being an integral part of the device.

### 2.5. Chemical and Phase Composition

Investigations of chemical composition and distribution of elements in coatings were performed using X-ray energy dispersion spectrometry (EDS) (Edax Inc., Mahwah, NJ, USA), an integral part of the scanning electron microscope (JEOL JSM-7800 F, JEOL Ltd. Tokio, Japan). The phases formed in the nanocomposite coating were investigated by X-ray diffraction (XRD) (XRD, Philips X’Pert Pro, Almelo, Netherlands). A monochromatic source, CuKα radiation (λ = 1.544 Å) was used, and the specimens were scanned from 20° to 90° at a scanning rate of 0.02°/s.

### 2.6. Mechanical Studies

Mechanical properties of the coatings were determined based on the nanoindentation test. The examination was carried out using a nanoindenter (NanoTest Vantage, Micro Materials, Wrexham, UK) equipped with a diamond, pyramidal Berkovich indenter with an apex angle of 124.4°. The coatings were subjected to 25 (5 × 5) nanoindentation measurements using identical parameters for each of the specimens. The maximum force was 5 mN, the loading and unloading rate were set up at 20 s, and the dwell period at maximum load was 10 s. The distance between individual indentations was 20 µm. The load–depth curve was obtained for each indentation. Nanohardness (H) reduced Young’s modulus (Er), and Young’s modulus (E) were determined using the Oliver–Phaar method and NanoTest software. A Poisson’s ratio of 0.25 was assumed for the coatings for converting reduced Young’s modulus into Young’s modulus, consider [89].

Nanoscratch tests were done with a nanoindenter (NanoTest Vantage, Micro Materials, Wrexham, UK). The coatings were subjected to 10 scratch tests with an increasing force from 0 to 200 mN at a distance of 500 μm and a loading rate of 1.3 mN/s. Based on the dependence of the friction force (Ft) on the normal force (Fn), NanoTest software was used to determine the critical friction load (Lf) and significant load (Lc) values causing the coating to peel off the substrate.

### 2.7. Contact Angle Studies

The measurement of the contact angle was conducted using a falling drop technique (Attention Theta Life, Biolin Scientific, Espoo, Finland) with a drop of distilled water at room temperature.

## 3. Results and Discussion

### 3.1. Morphology and Topography Studies

**Figure 1:**
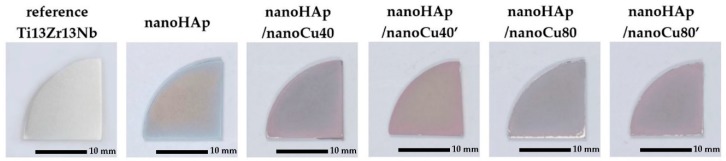
Macroscopic images of nanohydroxyapatite (nanoHAp) coating and nanoHAp coatings with copper nanoparticles.

Figure 1 shows macroscopic images of the specimens examined in this paper in their various forms: reference Ti13Zr13Nb alloy specimen after grinding; specimen with nanohydroxyapatite coating; specimens with nanohydroxyapatite and Cu nanoparticles coatings (nanoHAp/nanoCu40, nanoHAp/nanoCu40’, nanoHAp/nanoCu80, nanoHAp/nanoCu80’) after thermal treatment. Sintering of the coated specimens was conducted to increase the coating adhesion to the substrate, reduce its porosity, and change the crystal structure of nanoHAp from amorphous to crystalline [88].

In the macroscopic images, the color change of the surface of the coated specimens was compared to the surface of the reference specimen. In the case of nanohydroxyapatite coatings with Cu nanoparticles, the higher content of Cu nanoparticles in the electrolyte caused a darker, copper color of the specimen surface. All the produced coatings were characterized by high homogeneity, confirming the possibility of obtaining homogeneous nanohydroxyapatite coatings by the electrophoretic deposition methods, which was proven previously by Hadidi et al. [81].

**Figure 2:**
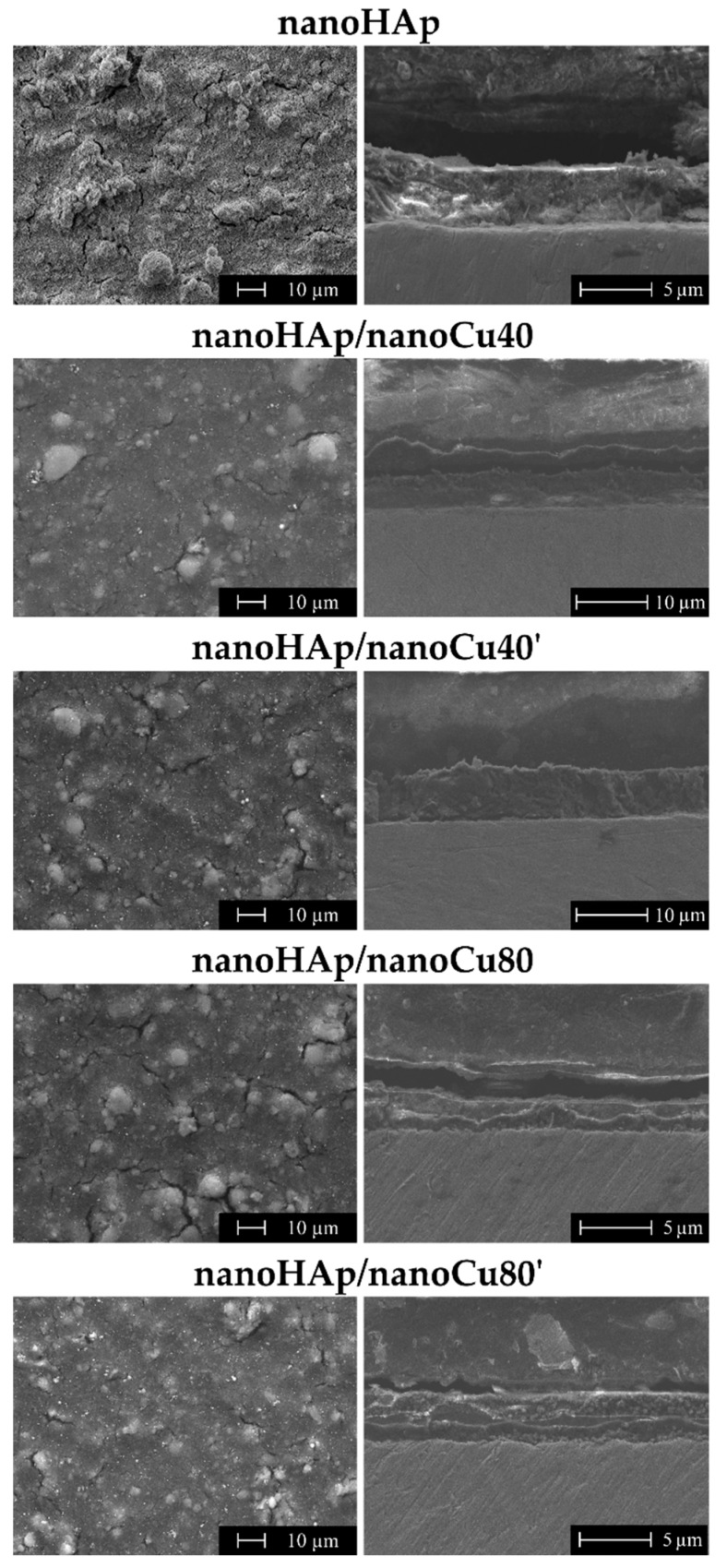
SEM images of the surface topography and cross-sections of the nanoHAp coating and nanoHAp coatings with copper nanoparticles (nanoCu).

Figure 2 shows SEM images of topographies and cross-sections of the nanohydroxyapatite coating and nanohydroxyapatite with Cu nanoparticles coatings obtained by electrophoretic deposition. 

The nanohydroxyapatite coatings were characterized by a relatively low number of cracks and agglomerates. The diameters of nanoHAp agglomerates ranged from a few to several dozen micrometers. Cu nanoparticles were visible on the surface of nanohydroxyapatite coatings of all specimens. The influence of the occurrence of metal nanoparticles in coatings on the quantity and size of cracks was observed. The appearance of Cu nanoparticles resulted in a decrease in the number of cracks on the surface of the coating compared to the nanoHAp coating, deposited with the same parameters, but in electrolyte without Cu nanoparticles. Thermal shrinkage of nanoHAp coatings during high-temperature heat treatment causes their cracking. The addition of Cu nanoparticles in coatings reduced the number of cracks as a result of the decrease of internal stresses appearing during thermal processing.

Moreover, the occurrence of nanoparticles can prevent the propagation of cracks in a hydroxyapatite-based coating [81]. In the case of a higher concentration of copper nanoparticles in electrolyte during deposition (80 nm), the increase in the concentration of Cu nanoparticles in the electrolyte additionally resulted in a reduction of the quantity and size of cracks. For smaller size of Cu nanoparticles (40 nm), the number and size of cracks on the coating surface for both groups (nanoHAp/nanoCu40 and nanoHAp/nanoCu40’) were similar. The research confirmed the positive effect of added metals in HAp coatings on the properties of coatings and same result was reported by Singh et al. [8,30,90]. For all nanohydroxyapatite coatings with Cu nanoparticles, the increasing concentration of copper nanoparticles in the electrolyte was accompanied by the increase of the number of copper nanoparticles’ agglomerates on the surface of coatings. The effect of the occurrence of Cu nanoparticles on the quantity of nanoHAp agglomerates on the surface of coatings was also observed. In the case of nanoHAp with nanoCu coatings, the smaller amount of agglomerates in comparison with nanoHAp coating may result from hindering the migration process of nanoHAp agglomerates due to much higher activity of nanometals than of nanoHAp particles and their larger size. In this case, only individual nanoparticles and smaller nanoHAp agglomerates were able to migrate correctly together with the metal nanoparticles. 

In the cross-sections of the nanohydroxyapatite coatings, no delamination between the coating and Ti13Zr13Nb substrate was observed. The thickness of the coatings and their Sa parameters have been presented in Table 3. Cracks and voids were not seen in the coating. Uneven coating thickness was found, which is a phenomenon characteristic for hydroxyapatite coatings obtained by electrophoretic deposition. Observed unevenness of the surface of the coatings was also confirmed by roughness measurements obtained with atomic force microscopy (AFM). The effects of the addition of copper nanoparticles and their contents in electrolyte on the delamination of coatings, as well as the occurrence of cracks and voids in the cross-sections in comparison to the nanohydroxyapatite coating without metal nanoparticles were not observed. Moreover, higher thickness of the coating translates into increased shrinking of material, which in turn causes more cracks.

For each of the coatings, significant deviations of coating thickness from average thickness values were noted due to their unevenness. In the case of nanohydroxyapatite coatings with Cu nanoparticles in nanoHAp/nanoCu80 and nanoHAp/nanoCu80’specimens, the thickness of the coatings was reduced as compared to the nanoHAp coating without nanometals. In the case of nanoHAp/nanoCu40 and nanoHAp/nanoCu40’ specimens, an increase in coating thickness was observed. For nanoHAp coatings with Cu nanoparticles, an increase of thickness accompanies the increase in the content of nanometals in the electrolyte.

**Figure 3:**
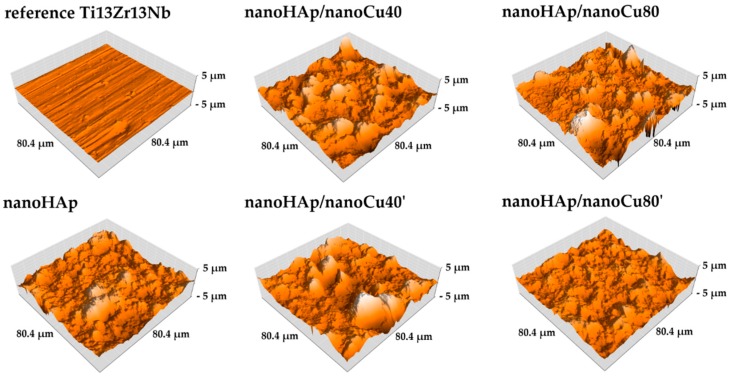
Atomic force microscopy (AFM) images of the surface topography of the reference Ti13Zr13Nb specimen, nanohydroxyapatite coating and nanohydroxyapatite coatings with nanoCu.

Figure 3 shows the topographies of the reference Ti13Zr13Nb specimen with nanohydroxyapatite coating and samples with nanohydroxyapatite coatings with nanoCu observed with atomic force microscopy (AFM). The obtained Sa parameters are presented in Table 3. An increase in surface roughness of the coated specimens compared to the surface of the reference Ti13Zr13Nb specimen was noted. Nanohydroxyapatite agglomerates were observed on the surfaces of all tested coatings. Obtained results of the AFM topography correlate with the results of the SEM examination. 

In the case of the conducted AFM measurements, the Sa parameter was derived as the mean of the individual 512 profilograms. Using a contactless AFM mode and a relatively long one-pass time (2 s), it was possible to obtain very accurate surface topography results. An increase was observed for all coating groups compared to the surface of the reference Ti13Zr13Nb specimen. In the case of coatings featuring agglomerates on the surface, a higher roughness value is noticeable. As for nanohydroxyapatite coatings with Cu nanoparticles, an increase in roughness was observed with the rise in nanoparticles content in the suspension, but only for smaller metal nanoparticles in the electrolyte. In coatings containing larger copper nanoparticles, the reported effect was reversed.

The surface roughness parameter, Sa, of the coatings used for implants is important in terms of osteoblast and bacteria adhesion. Moreover, its value determines the possibility of appropriate interaction of human tissues with the implant right in the initial phase after implant placement. A high level of roughness yields a better chance of tissue adhesion and primary stabilization between the implant and the bone [91,92]. Rough surfaces additionally facilitate the adhesion of a more significant number of osteoblasts and may stimulate the production of the extracellular matrix, therefore, in the case of implants, surfaces are designed in such a way as to increase this parameter [93,94]. The increase in roughness may also facilitate the adhesion of bacterial cells, which is an unfavorable phenomenon proved, among others, for *Staphylococcus epidermidis* [94].

### 3.2. Chemical and Phase Analysis

**Figure 4:**
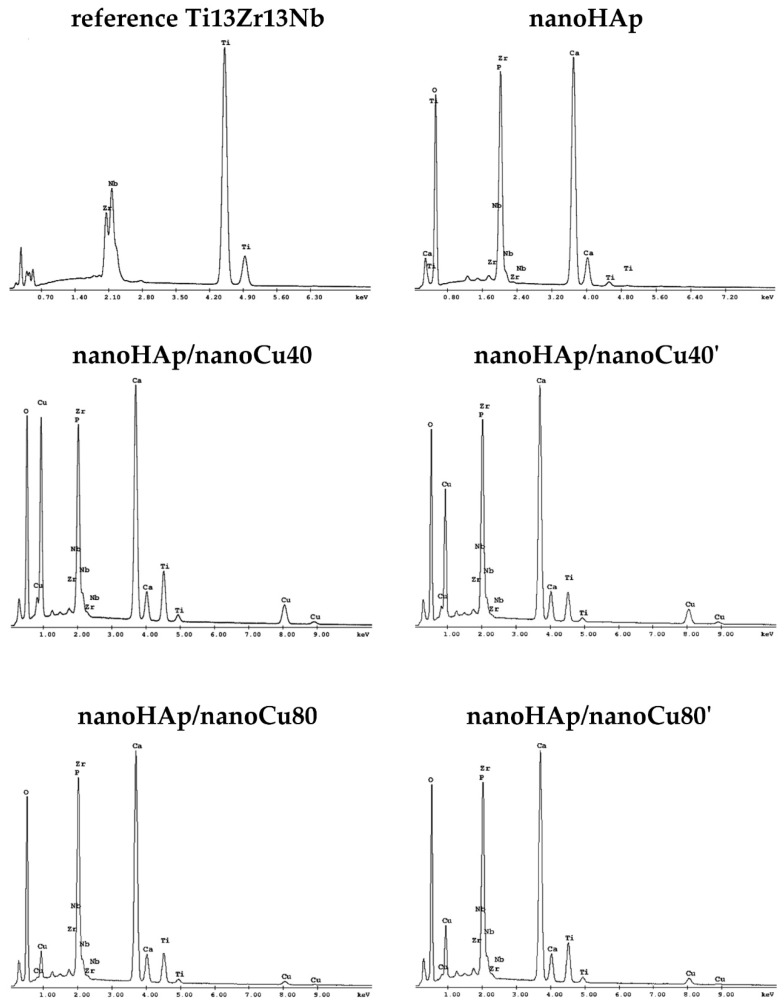
X-ray energy dispersion spectroscopy (EDS) spectra of reference specimens Ti13Zr13Nb, specimens with nanohydroxyapatite coatings, and specimens with nanohydroxyapatite coatings with nanoCu.

Figure 4 presents the results of X-ray energy dispersion spectroscopy (EDS) for the reference Ti13Zr13Nb specimen, specimens with nanohydroxyapatite coating, and specimens with nanohydroxyapatite coatings with nanocopper (nanoHAp/nanoCu40, nanoHAp/nanoCu40’, nanoHAp/nanoCu80, nanoHAp/nanoCu80’). In the EDS spectrum of the reference Ti13Zr13Nb specimen, the occurrence of three essential alloying elements: titanium, zirconium, and niobium was confirmed. The most intense peak was observed for titanium. In all the tested specimens with nanoHAp and nanoHAp with nanoCu coatings, constituents of the coating (O, Ca, P) and elements occurring in titanium alloy substrate (Ti, Zr, and Nb) were noted. The EDS results of nanoHAp with nanoCu coatings confirmed the presence of the nanoCu in coatings. 

In the case of nanohydroxyapatite coatings with Cu nanoparticles, the following elements of the coating are present: O, Ca, P, and Cu accompanied by Ti, Zr, and Nb originating from the substrate. Because of relatively thin coatings and the small number of metal nanoparticles in the tested coating, a small test area focused on the metallic nanoparticles visible on the surface of the coating was chosen to prove the occurrence of nanoCu.

**Figure 5:**
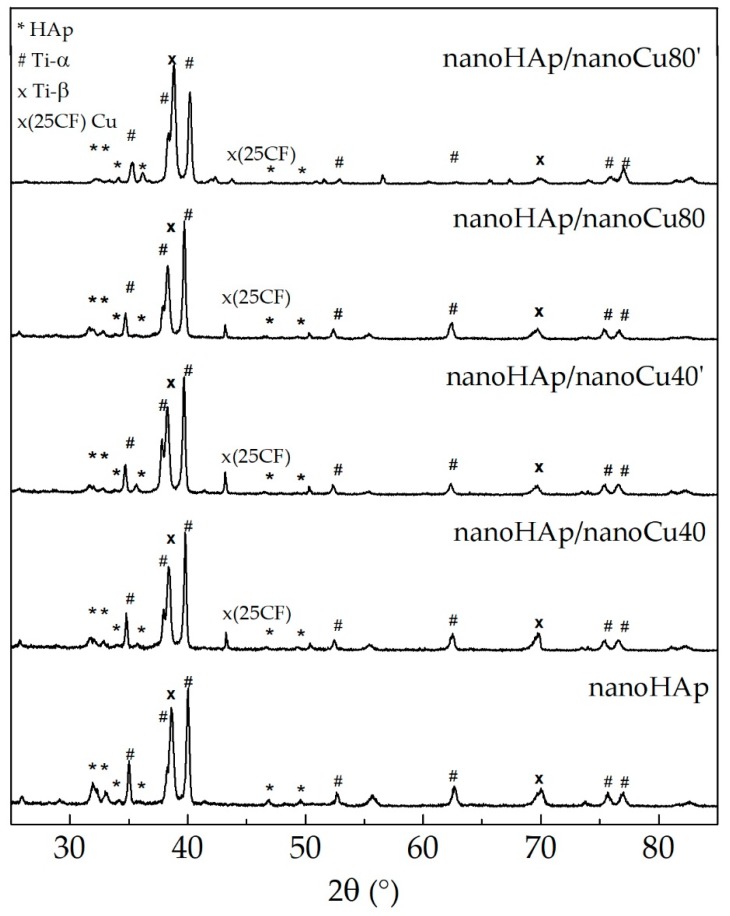
XRD diffractograms of nanohydroxyapatite coating and nanohydroxyapatite coatings with nanoCu.

Figure 5 presents X-ray diffractograms obtained for the specimens with nanohydroxyapatite coating and specimens with nanohydroxyapatite coating with Cu nanoparticles.

The main diffraction peaks observed in the diffractogram can be indexed as originating from the substrate alloy (Ti13Zr13Nb). As expected, they can be attributed to titanium. Moreover, the phase analysis shows that in the case of all investigated specimens, it is possible to index the peaks belonging to the hydroxyapatite phase and, in the case of specimens with a copper coating, to cubic copper as well. It was shown that the diffraction peaks observed at *2θ* at 25.9°, 31.8°, 32.2°, 32.9°, 34.1°, and 39.8° corresponded to the peaks of HAp [95]. In the specimens with a nanoHAp and nanocopper coating, the relative intensities of reflexes indexed as hydroxyapatite were lower in respect to the one attributed to the substrate. This can hint to a decreasing HAp layer thickness. The crystallinity of hydroxyapatite increases the bioactivity of the resulting coatings as the amorphous layer is unstable in the human body (it can dissolve within a few days after implantation), causing the loosening of an implant [96]. The coating procedure also influences the support crystal structure for nanoHAp/nanoCu80’ specimen, the change of the relative intensity of peaks originating from α and β titanium phases are visible. As previously reported [96], in the case of thermal treatment, the occurrence of hydroxyapatite impedes the phase transformation from α-Ti to β-Ti. On the other hand, the occurrence of niobium in the alloy should facilitate the process, as Nb is a β phase stabilizing element [97]. Therefore, one can assume that a treatment leading to the formation of copper layer also facilitates the phase transition between α and β titanium.

### 3.3. Nanomechanical Studies

**Figure 6:**
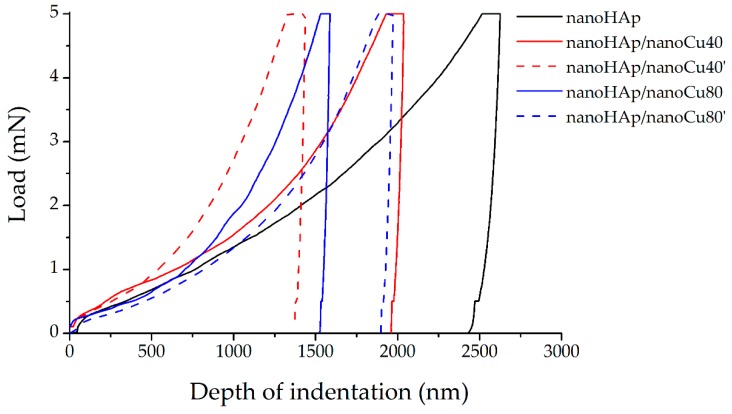
Hysteresis plots of load–deformation for a single indentation measurement for the nanohydroxyapatite and nanohydroxyapatite with nanoCu coatings.

The nanoindentation technique is a relatively new method used to study thin coatings and layers, including biomedical applications. Testing of mechanical properties, such as Young’s modulus and hardness, in thin specimens is not possible in some cases [31,98,99]. Figure 6 shows single hysteresis load–deformation graphs for nanohydroxyapatite coating (nanoHAp specimen) and nanohydroxyapatite coatings with nanoCu (nanoHAp/nanoCu40, nanoHAp/nanoCu40’, nanoHAp/nanoCu80, nanoHAp/nanoCu80’ specimens).

A single-load hysteresis plot consisting of three primary sections (force build-up, force dwell with maximum value, and offloading) was plotted during nanoindentation for each measurement. The shape of the unloading curve has a direct relation with the mechanical properties of the material determined by the Oliver–Pharr method [100]. In the case of the curves in Figure 6, the difference between the slope of the load curve and the deformation is clearly visible. Furthermore, the beginning of the deformation curve starts from the maximum depth of penetration and ends with the maximum depth value at which the indenter remains in contact with the test surface, referred to as the maximum contact depth. Small deflections occurring on the deformation curves are caused by the temperature drift performed with each measurement. 

The mechanical properties (nanohardness and Young’s modulus), nanoindentation properties (maximum indentation deflection), and nanoscratch test properties (critical load and critical friction) for all the tested specimens have been presented in Table 4.

In all nanoHAp coatings with nanoCu, an increase in nanohardness and Young’s modulus as compared with the nanoHAp coating was observed. Mechanical properties increase along with the rise in the number of copper nanoparticles. The highest values of nanohardness and Young’s modulus were reported for nanohydroxyapatite coating with nanoCu (specimen nanoHAp/nanoCu40’). The occurrence of metal nanoparticles in the coatings resulted in the rise of mechanical properties due to the increase of the homogeneity of coatings. Moreover, nanoCu in nanoHAp coatings acts as a reinforcing phase, preventing the propagation of cracks in the coatings [81].

In all coatings, the measurements showed that the lower value of maximum indentation depth was related to the higher value of nanohardness. Additionally, a significant value of the maximum depth deviation was noted for all the measurements. The value of plastic work in each case exceeded the value of elastic work. Moreover, the value of plastic work for all coatings increased with the increase of the maximum penetration depth of the indenter and, thus, with the decrease of the hardness and Young’s modulus.

Large deviations from the standard values of mechanical properties and nanoindentation properties shown in Table 4 can be attributed to the significant surface roughness of the coatings due to the occurrence of agglomerates and cracks on the surfaces. With a large number of individual measurements performed for each coating, it is an expected effect confirming the reliability and correctness of the performed tests. Reduced mechanical properties of the nanoHAp coatings can be attributed to the high porosity of the coatings, as well as to the impact of the shape and size of the nanoHAp powder particles. He and others [89] reported that an increase in the porosity of the coating caused deterioration of mechanical properties. Wei et al. [101] observed that the coniferous shape of HAp particles may cause higher susceptibility to cracking during shrinkage, followed by a decrease in mechanical properties. The low hardness of hydroxyapatite coatings is also attributed to the occurrence of large amounts of amorphous HAp in the structure. However, they were not detected in the tested coatings [102]. Drevet et al. [103] obtained similar results to the ones presented in this paper. However, they did not provide information on the thickness of the coatings obtained in the electrophoresis process nor the reasons for getting low mechanical properties. Mechanical properties of the coatings used for implants should have values similar to those for the cortical bone [104]. Unfortunately, coatings with poor mechanical properties can quickly dissolve in the human tissue environment [105].

**Figure 7:**
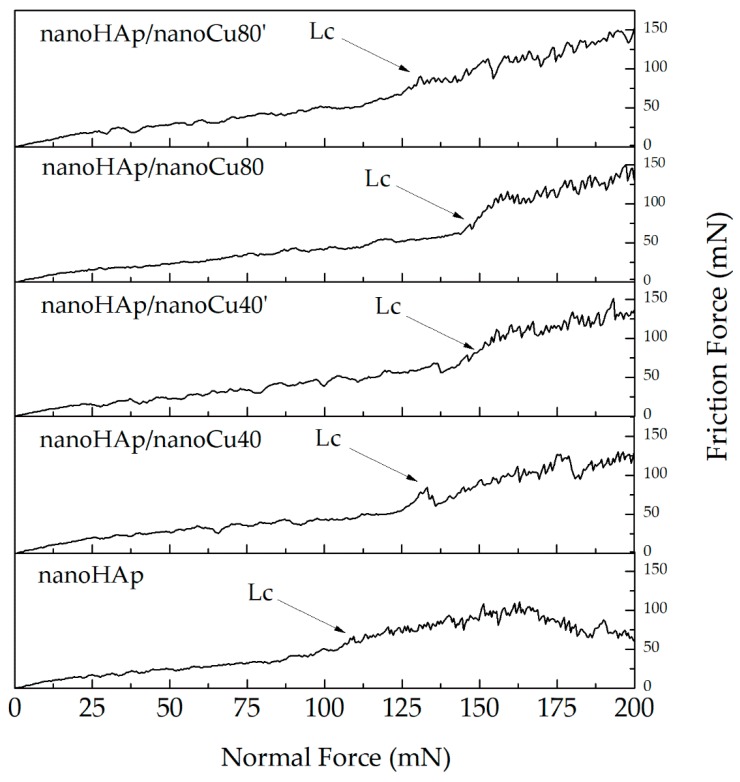
Plots of the friction force (Ft) as a function of normal force (Fn) with the critical force (Lc) of a single measurement for the nanohydroxyapatite and nanohydroxyapatite with nanocopper coatings.

Figure 7 shows plots of friction force (Ft) as a function of normal force (Fn) for a single nanoscratch measurement with a marked critical force (Lc) corresponding to the force of complete delamination of the nanohydroxyapatite coating from the Ti13Zr13Nb substrate. 

Sudden changes in the friction force were observed in all the results obtained. These changes determine the place of delamination of the coatings from the titanium substrate. The values of the critical load, Lc, determining the delamination force the coating from the substrate, and the critical friction force, Lf, determining the maximum value of the friction force at the beginning of the complete delamination of the coating from the metallic substrate for all tested nanohydroxyapatite coatings are shown in Table 4.

The addition of metallic nanoparticles in nanoHAp coatings increases the adhesion of the coatings to the metallic substrate. Among the nanoHAp with Cu nanoparticles coatings, the highest adhesion occurs for the nanoHAp/nanoCu80 specimen.

Suitable adhesion of hydroxyapatite coatings to a metal substrate is one of the most essential properties determining their quality. Detached particles of nanoHAp coatings can initiate inflammatory processes and bone atrophy around the implant [106]. The observed increase in the adhesion of nanoHAp coatings with nanometals can be attributed to the higher homogeneity of these coatings. Additionally, nanoHAp coatings with nanometals can create metallic bonds between nanocopper in the coating and the substrate materials observed in [81]. High values of the obtained standard deviations are caused by uneven thickness of the tested coatings and also by the occurrence of agglomerates and cracks on their surface.

### 3.4. Contact Angle Measurements

Table 5 shows the values of the average contact angle for the reference Ti13Zr13Nb specimen and specimens with nanohydroxyapatite coatings.

The obtained results of the average contact angle measurements (left and right) confirmed the hydrophilic character of both reference material Ti13Zr13Nb and all the tested coatings. The hydrophilic nature of the surface is a feature required from the materials used for implants to improve osseointegration of the implant with bone [107]. All specimens with nanohydroxyapatite coatings with large nanoparticles can prevent liquid from entering the coating. Therefore the value of the contact angle was much higher than in the case of other nanoHAp coatings with nanometals. All tested coatings had an average contact angle smaller than the reference specimen Ti13Zr13Nb which was due to their porous structure allowing the liquid to penetrate deep into the coating. In the case of nanoHAp coatings with nanometals, the obtained low contact angle values may result from the higher wettability of metals as opposed to ceramic materials. In the case of nanoHAp/nanoCu’80, an extensively high concentration of relatively large nanoparticles can prevent liquid from entering the coating. Therefore the contact angle is much higher than that of other nanoHAp coatings with nanometals. Huang et al. reported that the most appropriate surface contact angle that can promote cell attachment is 20°–40° [51].

The problem of harmful effects of various bacteria on the implant early fixation and lifetime was examined, for example, in dentistry based on clinical experience [108], and nanotechnologies as a valuable tool in fighting bacteria were already suggested [109]. However, the proposed solution, nanohydroxyapatite with incorporated nanocopper, needs further in vitro and in vivo assays to be implemented in clinical practice.

## 4. Conclusions

The electrophoretic method allows the production of nanohydroxyapatite coatings and the simultaneous production of a composite nanohydroxyapatite with nanocopper coatings on Ti13Zr13Nb titanium alloy, whose properties significantly depend on the applied process parameters. The size and content of copper nanoparticles are essential the process parameters. Too large nanoparticles disturb the migration of particles into the forming coating and cause a decrease in its homogeneity. The excessively high concentration of nanoparticles has a similar effect. The addition of nanocopper prevents the propagation of cracks arising as a result of thermal stresses after heat treatment. Coatings characterized by higher packing of nanoparticles adhere better to the titanium substrate which can be attributed to the decreasing porosity of the coating and its increasing homogeneity. The increase in the porosity of the structure and the occurrence of metallic nanoparticles cause a decrease in the contact angle and, consequently, an increase in biocompatibility and bioactivity.

## Figures and Tables

**Table 1:** The chemical composition of the Ti13Zr13Nb alloy, wt.% (according to the manufacturer’s attestation).

Element	Nb	Zr	Fe	C	N	O	H	Ti
wt.%	13.5	13.5	0.05	0.04	0.013	0.11	0.04	remainder

**Table 2:** Designations of experiment specimens with the characteristic process parameters.

Specimen	Properties of Electrophoretic Deposition				
	NanoHAp Content/100 mL of Ethanol (g)	Average Particle Size of NanoCu Powder (nm)	NanoCu Content/100 mL of Ethanol (g)	Voltage of Deposition (V)	Time of Deposition (min)
nanoHAp	0.1	–	–	30	2
nanoHAp/nanoCu40		40	0.01		
nanoHAp/nanoCu40’			0.025		
nanoHAp/nanoCu80		80	0.01		
nanoHAp/nanoCu80’			0.025		

**Table 3:** The thickness and surface roughness (Sa) parameters of the reference specimen and specimens with nanoHAp and nanoHAp with nanoCu coatings.

Specimen	Properties	
	Thickness (µm)	Sa Parameters (µm)
Reference Ti13Zr13Nb	–	0.13
nanoHAp	4.67 ± 1.07	0.64
nanoHAp/nanoCu40	6.27 ± 1.48	0.73
nanoHAp/nanoCu40’	7.74 ± 1.45	0.86
nanoHAp/nanoCu80	2.42 ± 0.34	0.76
nanoHAp/nanoCu80’	3.28 ± 0.31	0.44

**Table 4:** Mechanical, nanoindentation, and nanoscratch test properties of nanohydroxyapatite coatings.

Properties	Nanoindentation Properties				Nanoscratch Test Properties	
Specimen	Nanohardness (GPa)	Young’s Modulus, E (GPa)	Maximum Depth of Indentation (nm)	E^3^/h^2^ (GPa)	Critical Load, Lc (mN)	Critical Friction, Lf (mN)
nanoHAp	0.032 ± 0.009	4.46 ± 0.91	2617.12 ± 359.26	86.64	106.77 ± 37.51	59.18 ± 20.46
nanoHAp/nanoCu40	0.054 ± 0.020	10.27 ± 3.16	2084.71 ± 382.15	371.47	123.84 ± 52.46	59.14 ± 24.18
nanoHAp/nanoCu40’	0.139 ± 0.050	17.82 ± 5.48	1287.28 ± 279.89	292.88	141.89 ± 13.09	78.99 ± 10.02
nanoHAp/nanoCu80	0.051 ± 0.026	10.51 ± 3.63	1887.62 ± 479.95	446.34	155.24 ± 12.78	94.47 ± 9.73
nanoHAp/nanoCu80’	0.059 ± 0.038	11.86 ± 6.09	2224.33 ± 757.61	479.24	128.73 ± 30.39	80.39 ± 21.01

**Table 5:** The value of the average contact angle for the reference specimen and specimens with nanohydroxyapatite coatings.

Specimen	Average Contact Angle (°)
Reference Ti13Zr13Nb	53.7 ± 2.1
nanoHAp	35.8 ± 3.5
nanoHAp/nanoCu40	22.6 ± 2.2
nanoHAp/nanoCu40’	18.2 ± 1.9
nanoHAp/nanoCu80	26.7 ± 2.8
nanoHAp/nanoCu80’	48.3 ± 2.5
